# CMR characterization of patients with heart failure and left bundle branch block

**DOI:** 10.1093/ehjimp/qyae047

**Published:** 2024-05-17

**Authors:** Raluca Tomoaia, Peter Harrison, Lydia Bevis, Ali Wahab, Patrick Thompson, Christopher E D Saunderson, Eylem Levelt, Erica Dall’Armellina, Pankaj Garg, John P Greenwood, Sven Plein, Peter P Swoboda

**Affiliations:** Biomedical Imaging Sciences Department, Leeds Institute of Cardiovascular and Metabolic Medicine, University of Leeds, Woodhouse, Leeds, West Yorkshire LS2 9JT, UK; Cardiology Department, Iuliu Hatieganu University of Medicine and Pharmacy, Cluj-Napoca, Romania; Biomedical Imaging Sciences Department, Leeds Institute of Cardiovascular and Metabolic Medicine, University of Leeds, Woodhouse, Leeds, West Yorkshire LS2 9JT, UK; Biomedical Imaging Sciences Department, Leeds Institute of Cardiovascular and Metabolic Medicine, University of Leeds, Woodhouse, Leeds, West Yorkshire LS2 9JT, UK; Biomedical Imaging Sciences Department, Leeds Institute of Cardiovascular and Metabolic Medicine, University of Leeds, Woodhouse, Leeds, West Yorkshire LS2 9JT, UK; Biomedical Imaging Sciences Department, Leeds Institute of Cardiovascular and Metabolic Medicine, University of Leeds, Woodhouse, Leeds, West Yorkshire LS2 9JT, UK; Biomedical Imaging Sciences Department, Leeds Institute of Cardiovascular and Metabolic Medicine, University of Leeds, Woodhouse, Leeds, West Yorkshire LS2 9JT, UK; Biomedical Imaging Sciences Department, Leeds Institute of Cardiovascular and Metabolic Medicine, University of Leeds, Woodhouse, Leeds, West Yorkshire LS2 9JT, UK; Biomedical Imaging Sciences Department, Leeds Institute of Cardiovascular and Metabolic Medicine, University of Leeds, Woodhouse, Leeds, West Yorkshire LS2 9JT, UK; Department of Cardiovascular and Metabolic Health, Norwich Medical School, University of East Anglia, Norfolk, UK; Biomedical Imaging Sciences Department, Leeds Institute of Cardiovascular and Metabolic Medicine, University of Leeds, Woodhouse, Leeds, West Yorkshire LS2 9JT, UK; Biomedical Imaging Sciences Department, Leeds Institute of Cardiovascular and Metabolic Medicine, University of Leeds, Woodhouse, Leeds, West Yorkshire LS2 9JT, UK; Biomedical Imaging Sciences Department, Leeds Institute of Cardiovascular and Metabolic Medicine, University of Leeds, Woodhouse, Leeds, West Yorkshire LS2 9JT, UK

**Keywords:** heart failure, left bundle branch block, magnetic resonance imaging

## Abstract

**Aims:**

We aimed to identify the distinctive cardiovascular magnetic resonance (CMR) features of patients with left bundle branch block (LBBB) and heart failure with reduced ejection fraction (HFrEF) of presumed non-ischaemic aetiology. The secondary aim was to determine whether these individuals exhibit characteristics that could potentially serve as predictors of left ventricular ejection fraction (LVEF) recovery as compared with patients without LBBB.

**Methods and results:**

We prospectively recruited patients with HFrEF (LVEF ≤ 40%) on echocardiography who were referred for early CMR examination. Patients with an established diagnosis of coronary artery disease and known structural or congenital heart disease were excluded. LV recovery was defined as achieving ≥10% absolute improvement to ≥40% in LVEF between baseline evaluation to CMR. A total of 391 patients were recruited including 115 (29.4%) with LBBB. Compared with HF patients without LBBB, those with LBBB exhibited larger left ventricles and smaller right ventricles, but no differences were observed with respect to LVEF (35.8 ± 12 vs. 38 ± 12%, *P* = 0.105). The overall rate of LV recovery from baseline echocardiogram to CMR (70 [42–128] days) was not significantly different between LBBB and non-LBBB patients (27.8% vs. 31.5%, *P* = 0.47). Reduced LVEF remained an independent predictor of LV non-recovery only in patients with LBBB.

**Conclusion:**

Patients presenting with HFrEF and LBBB had larger LV cavities and smaller RV cavities than those without LBBB but no difference in prevalence of scar or ischaemia. The rates of LV recovery were similar between both groups, which supports current guidelines to defer device therapy until 3–6 months of guideline-directed medical therapy, rather than early CMR and device implantation.

## Graphical Abstract

**Graphical Abstract:**
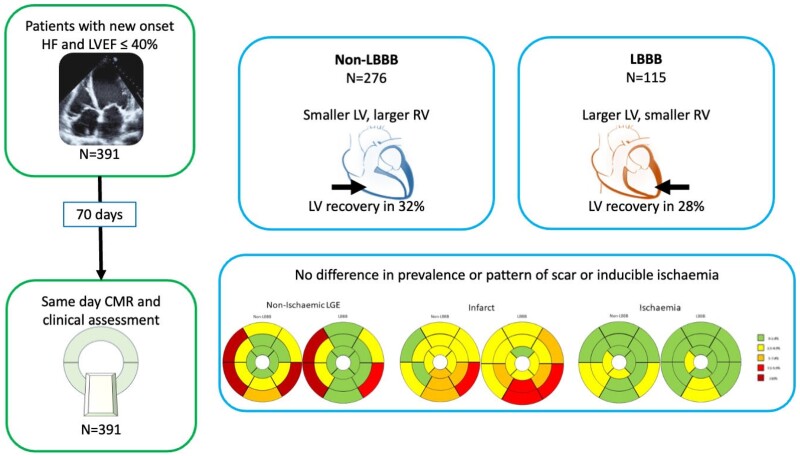

## Introduction

Several studies have focused on the characteristics of patients with heart failure and reduced ejection fraction (HFrEF) in an effort to identify those at high risk or who might benefit from cardiac device therapy. Cardiovascular magnetic resonance (CMR) is recommended in international guidelines for tissue characterization, volumetric assessment (particularly in those with poor echocardiographic windows), and assessment of prior myocardial infarction.^1^ Previous work indicates that non-ischaemic aetiology, shorter duration of HF, female sex, lack of myocardial scar, and initial left ventricular ejection fraction (LVEF) are predictive factors for LV recovery in patients with HFrEF.^2,3^ Furthermore, late gadolinium enhancement (LGE) has identified ischaemic scar in patients with HF who are assumed to have a non-ischaemic aetiology.^4,5^

Patients with left bundle branch block (LBBB) form a distinct subgroup among individuals with HF, since their clinical features are influenced by abnormal myocardial activation. These patients have increased likelihood of a favourable response to cardiac resynchronization therapy (CRT).^1^ Nonetheless, there are limited data available to accurately determine whether LBBB is associated with a distinct morphological or functional phenotype as defined by CMR, such as tissue characteristics and left ventricular scar.

With advances in the medical therapy of heart failure, approximately one-third of patients with HFrEF experience LVEF recovery.^6,7^ Presently, clinical guidelines therefore advise initiating guideline-directed medical therapy (GDMT) for a duration of three to six months prior to planning CRT implantation.^1^ Nevertheless, the true incidence of patients achieving LVEF recovery with GDMT varies across different studies and the proportion of patients with LBBB who experience recovery is even less well-defined.^6^ This may be attributed, in part, to the varying definitions of HF with improved ejection fraction (HFimpEF) across studies, but also to the limited characterization by imaging of these patients.

The aim of this study was to identify the CMR characteristics in patients with LBBB and HFrEF to determine whether these individuals exhibit distinct features that may predict LVEF recovery, as compared with patients without LBBB.

## Methods

### Study population

Participants from the MATCH heart failure registry (MyocArdial Tissue CHaracteristics in patients with heart failure) were prospectively enrolled in the study. These were patients that were recently diagnosed with heart failure and referred for CMR examination from February 2018 to August 2023. The exclusion criteria consisted of one or more of the following: LVEF > 40% at initial evaluation, an established diagnosis of coronary artery disease (CAD) (significant CAD defined as >70% stenosis in a major coronary artery or >50% of the left main on coronary angiography, previous myocardial infarction, history of myocardial revascularization, or typical angina), known presence of structural heart disease (hypertrophic cardiomyopathy, amyloidosis), suspected myocarditis, congenital heart disease, and significantly impaired renal function.

On the same day as the CMR, patients underwent clinical evaluation. Comorbidities and previous medications were either collected directly or by accessing the electronic health record. The LVEF, as determined at the initial echocardiographic evaluation, was obtained for all patients according to ASE criteria.^8^ LV recovery was defined as achieving ≥10% absolute improvement to a level of 40% or greater of LVEF from baseline evaluation upon the date of CMR quantification.^1,7^ Following identification of HF, guideline-directed medical therapy (GDMT) was initiated based on the decision of the treating physician.

Electrocardiograms were subsequently analysed to establish the presence or absence of LBBB, which was defined as per the criteria provided by the European Society of Cardiology.^9^

HFimpEF was defined in accordance with the latest ESC recommendations (LVEF > 40% at initial evaluation with ≥10% absolute improvement to a level of 40% or greater from baseline evaluation).^1^

### CMR acquisition

The CMR study was conducted using a 3 T system (Siemens Magnetom Prisma, Erlangen, Germany). It was recommended that patients avoid caffeine for at least 24 h preceding the CMR study. The study protocol included the following acquisitions: (i) long- and short-axis cine sequences, (ii) adenosine stress perfusion imaging, and (iii) motion-corrected bright-blood LGE with phase-sensitive inversion-recovery sequences in both long- and short-axis orientation, with additional dark-blood LGE images in cases where necessary if the presence of a subendocardial scar was uncertain.

Adenosine infusion at a dose of 140 g/kg/min was performed for a minimum of 3 min for stress imaging. In the event of inadequate haemodynamic response (heart rate increase of <10 beats per min) or absence of symptomatic response, the infusion rate was increased progressively up to 210 μg/kg/min.

### Image analysis

Image processing and analysis were conducted using the cvi42 software (Circle Cardiovascular Imaging, Calgary, Alberta, Canada). Volumes and mass were obtained by delineation of endocardial and epicardial borders in short-axis (ventricles) and long-axis (atria) cine images at the end of systole and diastole, excluding trabeculations. All the measurements were indexed to body surface area.

Inducible ischaemia was defined by the presence of a perfusion defect on visual analysis affecting two or more myocardial segments at stress, which was not apparent at rest perfusion and with no matching ischaemic scar on LGE imaging.

LGE was defined by the presence of visual enhancement in at least one segment, in either two perpendicular planes or in both bright and black blood images. Subendocardial enhancement with a coronary distribution was defined as ischaemic LGE. All other patterns of fibrosis were categorized as non-ischaemic, with the exception of fibrosis at the right ventricular insertion points of the interventricular septum that was not reported.

### Statistical analysis

Clinical variables were reported as either mean ± SD/median [Q1–Q3] or frequencies depending on the type and distribution of the data. The Kolmogorov–Smirnov test was used to assess normality. Patients were categorized into two groups based on the presence or absence of LBBB. The continuous demographic, clinical, echocardiographic, and CMR characteristics were compared using *t*-tests for normally distributed data, and the Mann-Whitney test otherwise. The χ^2^ test was used for categorical data.

Inducible ischaemia and ischaemic and non-ischaemic LGE were graphically represented as percentage of positives within each segment of the myocardium, using the AHA 16 segment model.

Univariate logistic regression was conducted to analyse the relationship between the LVEF recovery and both clinical, echocardiographic, and CMR-assessed variables in each group. Multivariate logistic regression was performed to determine whether the initial LVEF evaluation and ischaemic LGE could independently predict LV recovery while adjusting for clinical parameters by the backward method.

Statistical analysis was conducted using MedCalc Statistical Software 19.6.1 (MedCalc Software Ltd, Ostend, Belgium; http://www.medcalc.org). A *P* value of <0.05 was considered significant.

## Results

### Patients

**Figure 1:**
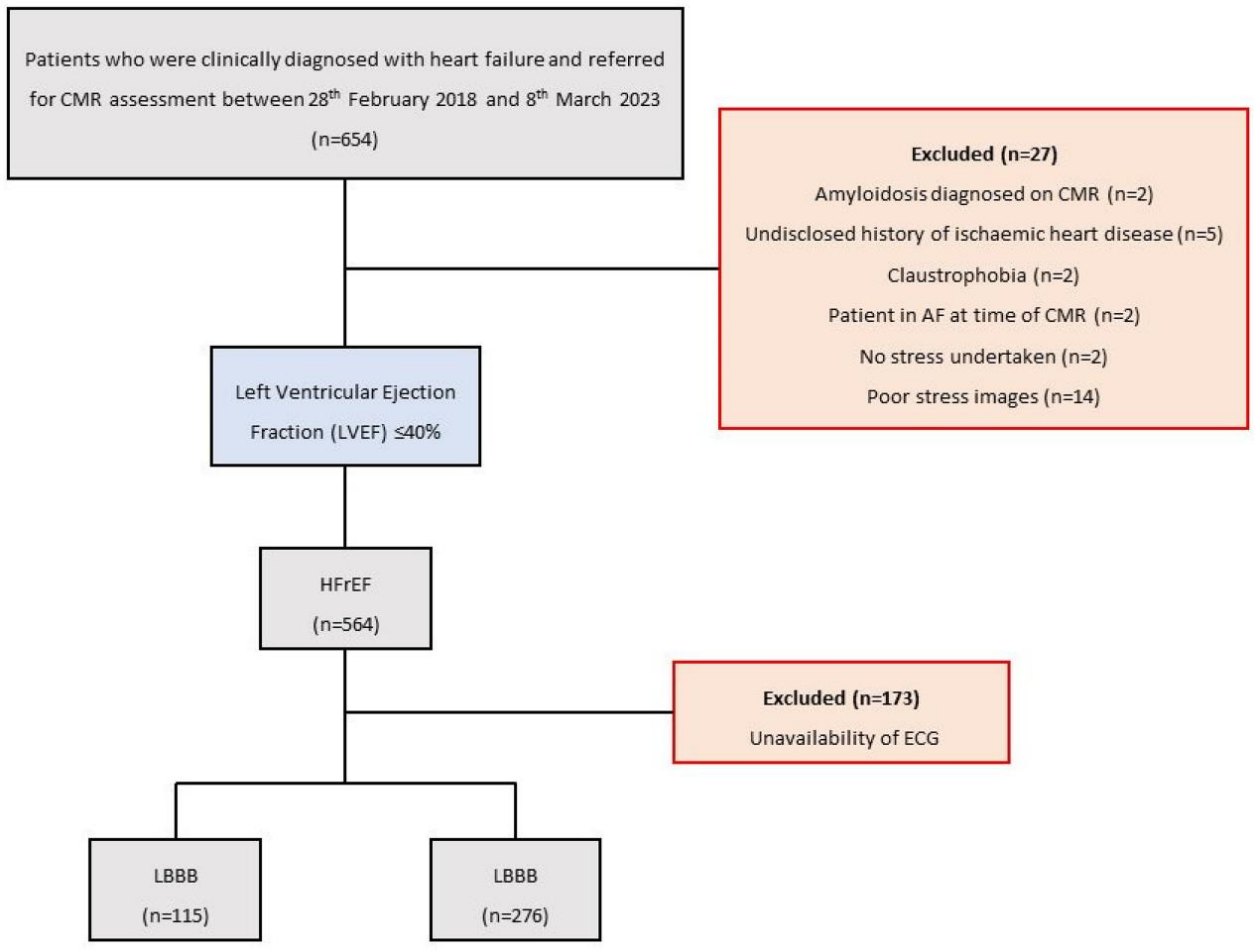
CONSORT diagram. CMR, cardiac magnetic resonance; CONSORT, Consolidated Standards of Reporting Trials; ECG, electrocardiogram; HFrEF, heart failure with reduced left ventricular ejection fraction; LBBB, left bundle branch block; LVEF, left ventricular ejection fraction.

A total of 391 patients of the initial 654 patients in the MATCH registry were included in this study. A total of 263 patients were excluded due to unavailability of the ECG (*n* = 173), amyloidosis diagnosed on CMR (*n* = 2), undisclosed history of ischaemic heart disease (*n* = 5), claustrophobia (*n* = 2), atrial fibrillation at time of CMR (*n* = 2), no stress undertaken (*n* = 2), poor stress images (*n* = 14), and baseline LVEF > 40% (*n* = 63). Among these individuals, 115 (29.4%) had LBBB (*Figure 1*).

### Clinical characteristics, echocardiography, medication, and biomarkers

The mean age of the patients was 63.4 ± 12.2 years, and 263 (67.3%) were male. *Table 1* shows the differences in baseline characteristics between patients with LBBB and those without. Patients with LBBB were older than those without (66.4 ± 12.3 vs. 62.2 ± 12 years, *P* = 0.0017). Regarding comorbidities and cardiovascular risk factors, LBBB patients were comparable to non-LBBB individuals, apart from atrial fibrillation, which was less prevalent in those with LBBB (20.8 vs. 43.8%, *P* = 0.0001). No significant differences were observed between groups in terms of diabetes mellitus, hypertension, hypercholesterolaemia, previous cerebrovascular events, or smoking history.

**Table 1:** Baseline characteristics

	All subjects (*n* = 391)	LBBB (*n* = 115)	Non-LBBB (*n* = 276)	*P* value
Clinical parameters				
Age (years)	63.4 ± 12.2	66.4 ± 12.3	62.2 ± 12	0.0017
Sex (male)	263 (67.3)	69 (60)	194 (70.3)	0.043
Heart rate (bpm)	71 ± 15	69 ± 14	72 ± 15	0.13
Systolic blood pressure (mmHg)	123 ± 20	126 ± 21	121 ± 19	0.035
Diastolic blood pressure (mmHg)	74 ± 10	73 ± 10	75 ± 10	0.23
QRS duration (ms)	115 ± 30	151 ± 18	99 ± 19	<0.0001
NYHA class > II, *n* (%)	36 (9.2)	10 (8.7)	26 (9.4)	0.63
Oedema, *n* (%)	53 (13.6)	21 (18.3)	32 (11.6)	0.08
Orthopnoea, *n* (%)	70 (17.9)	15 (13)	55 (19.9)	0.11
Shortness of breath on exertion, *n* (%)	191 (48.8)	59 (51.3)	132 (47.8)	0.68
Comorbidities				
Diabetes mellitus, *n* (%)	70 (17.9)	20 (17.4)	50 (18.1)	0.09
Hypertension, *n* (%)	173 (44.2)	58 (50.4)	115 (41.7)	0.11
Hypercholesterolaemia, *n* (%)	106 (27.1)	39 (33.9)	67 (24.3)	0.051
Atrial fibrillation, *n* (%)	147 (37.6)	26 (20.8)	121 (43.8)	0.0001
Stroke, *n* (%)	54 (13.8)	20 (22.6)	34 (12.3)	0.19
Smoking				
Current smoker, *n* (%)	63 (16.1)	16 (13.9)	47 (17)	0.44
Ex-smoker, *n* (%)	154 (39.4)	48 (41.7)	106 (38.4)	0.56
Medications				
Antiplatelets, *n* (%)	76 (19.4)	32 (27.8)	44 (15.9)	0.047
Beta-blocker, *n* (%)	338 (86.4)	97 (84.3)	241 (87.3)	0.08
Statin, *n* (%)	180 (46)	62 (53.9)	118 (42.8)	0.009
ACE-I/ARB, *n* (%)	337 (86.2)	98 (85.2)	239 (86.6)	0.09
Sacubitril/valsartan, *n* (%)	25 (6.4)	5 (4.3)	20 (7.2)	0.053
Aldosterone-receptor antagonist, *n* (%)	155 (39.6)	42 (36.5)	113 (40.9)	0.07
SGLT2i, *n* (%)	41 (10.5)	11 (9.6)	30 (10.9)	0.76
Diuretic, *n* (%)	183 (46.8)	45 (38.2)	138 (50)	0.017
Oral anti-glycaemic agent, *n* (%)	70 (17.9)	20 (17.4)	50 (18.1)	0.09
Oral anticoagulant, *n* (%)	139 (35.5)	27 (23.5)	112 (40.6)	0.0007
Echocardiographic evaluation				
Initial LVEF at echocardiography (%)	29 ± 9	30 ± 7	29 ± 9	0.3
Duration echocardiography—CMR (days)	70 [42–128]	74 [42.8–134.3]	70 [40.3–126]	0.53
LVEF increase between evaluations (%)	8.4 [0.85–15.3]	6.2 [−1.25–12.9]	8.8 [1.9–15.7]	0.03
Recovery of LVEF, *n* (%)	123 (31.4)	32 (27.8)	87 (31.5)	0.47

ACE-I, angiotensin-converting enzyme inhibitor; ARB, angiotensin receptor blocker; CMR, cardiac magnetic resonance; LBBB, left bundle branch block; LVEF, left ventricular ejection fraction.

Regarding GDMT, antiplatelet (27.8% vs. 15.9%, *P* = 0.047) and statin therapy (53.9% vs. 42.8%, *P* = 0.009) were more commonly used in LBBB patients, whereas diuretics (38.2% vs. 50%, *P* = 0.017) and oral anticoagulants (23.5% vs. 40.6%, *P* = 0.0007) were administered less frequently in this group.

Compared with non-LBBB patients, those with LBBB had slightly higher LVEF on initial assessment, but the difference did not reach statistical significance (30 ± 7 vs. 29 ± 9%, *P* = 0.3). In contrast, the increase in LVEF between evaluations was more evident in the non-LBBB group (8.8 [1.9–15.7] vs. 6.2 [−1.25–12.9], *P* = 0.03). A proportion of both LBBB (*n* = 32, 27.8%) and non-LBBB patients (*n* = 87, 31.5%) demonstrated LV recovery from baseline echocardiography evaluation and CMR (70 [42–128] days) while receiving GDMT, but the overall rate of LV recovery was not significantly different between the two groups (*P* = 0.47).

### CMR evaluation

The differences in CMR parameters between patients with and without LBBB are depicted in *Table 2*. LBBB patients demonstrated larger left ventricular dimensions (LVEDVi of 124.5 ± 40.5 vs. 108.9 ± 36.7 mL/m^2^, *P* = 0.0002, LVESVi of 83.3 ± 40 vs. 70.6 ± 35.8 mL/m^2^, *P* = 0.0039) but smaller right ventricular dimensions (RVEDVi of 73.8 ± 23.8 vs. 80.2 ± 25.1 mL/m^2^, *P* = 0.021 and RVESVi of 36.1 ± 20.2 vs. 44.3 ± 16 mL/m^2^, *P* = 0.0004). However, no differences were observed with respect to LVEF (35.8 ± 12 vs. 38 ± 12%, *P* = 0.21). RVEF was more decreased in patients without LBBB (47.5 ± 23 vs. 53 ± 12%, *P* = 0.02).

**Table 2:** Clinical CMR parameters

	All subjects (*n* = 391)	LBBB (*n* = 115)	Non-LBBB (*n* = 276)	*P* value
Left ventricular wall thickness (mm)	84 ± 20	83 ± 20	84 ± 20	0.47
Left ventricular end-diastolic volume (mL)	223 ± 76	243.2± 83	214.8 ± 71.6	0.0007
Left ventricular end-diastolic volume index (mL/m^2^)	113.5 ± 38.5	124.5 ± 40.5	108.9 ± 36.7	0.0002
Left ventricular end-systolic volume (mL)	146.3 ± 73	162.7 ± 79.4	139.4 ± 69.1	0.0039
Left ventricular end-systolic volume index (mL/m^2^)	74.4 ± 38	83.3 ± 40	70.6 ± 35.8	0.0023
Left ventricular ejection fraction	37 ± 12.3	35.8 ± 12	38 ± 12	0.21
Left ventricular mass (g)	136.4 ± 43.1	142.9 ± 40.4	134 ± 44	0.056
Left ventricular mass index (g/m^2^)	69 ± 20	73 ± 18.6	67.3± 20.3	0.025
Right ventricular end-diastolic volume (mL)	155.5 ± 54.7	146.6 ± 53.8	159.2 ± 54.7	0.04
Right ventricular end-diastolic volume index (mL/m^2^)	78.3 ± 24.8	73.8 ± 23.8	80.2 ± 25.1	0.021
Right ventricular end-systolic volume (mL)	83.7 ± 45	72.4 ± 44.6	88.4 ± 44.4	0.0014
Right ventricular end-systolic volume index (mL/m^2^)	41.9 ± 21.2	36.1 ± 20.2	44.3 ± 16	0.0004
Right ventricular ejection fraction (%)	49 ± 20.4	53 ± 12	47.5 ± 23	0.02
Left atrial volume index (mL/m^2^)	44.1 ± 19	42.5 ± 18.7	44.8 ± 19.2	0.27
Ischaemic LGE, *n* (%)	79 (20.2)	21 (18.3)	58 (21)	0.54
Inducible ischaemia, *n* (%)	28 (7.2)	7 (6.1)	21 (7.6)	0.6
Non-ischaemic LGE, *n* (%)	119 (30.4)	29 (25.2)	90 (32.6)	0.15
Ischaemic LGE extent, *n* (%)				0.59
No LGE		94 (81.7)	221 (80.1)	
1–2 segments		8 (7)	26 (9.4)	
3–4 segments		3 (2.6)	10 (3.6)	
5–6 segments		5 (4.3)	10 (3.6)	
>6 segments		5 (4.3)	9 (3.3)	
Non-ischaemic LGE extent, *n* (%)				0.91
No LGE		86 (74.8)	191 (69.2)	
1–2 segments		23 (20)	57 (20.7)	
3–4 segments		4 (3.5)	18 (6.5)	
5–6 segments		1 (0.9)	4 (1.4)	
>6 segments		1 (0.9)	6 (2.2)	

LBBB, left bundle branch block; LGE, late gadolinium enhancement.

**Figure 2:**
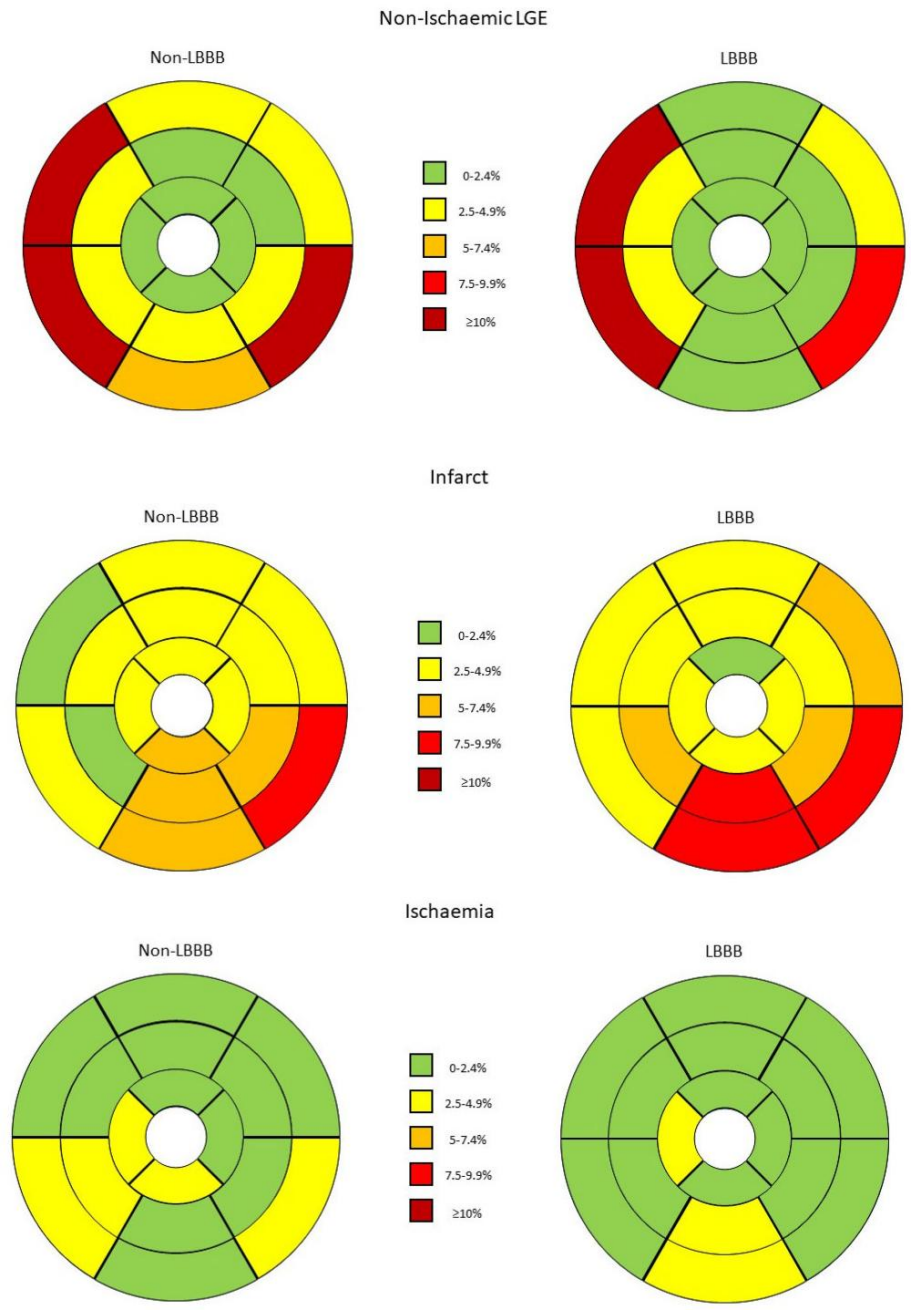
Distribution of segmental non-ischaemic LGE, ischaemic LGE, and inducible ischaemia in patients with LBBB and those without. LBBB, left bundle branch block; LGE, late gadolinium enhancement.

The prevalence of ischaemic scar (18.3% vs. 21%, *P* = 0.54) and non-ischaemic fibrosis (25.2% vs. 32.6%, *P* = 0.15) was comparable in patients with LBBB and controls. Furthermore, apart from segment 4, which presented non-ischaemic fibrosis less frequently in LBBB patients (0.9% vs. 6.2%, *P* = 0.023), no difference was observed with respect to the prevalence of non-ischaemic or ischaemic LGE in specific segments between LBBB and non-LBBB patients (*Figure 2*).

Inducible ischaemia was seen in 6.1% of LBBB patients and in 7.6% of non-LBBB individuals, with no significant difference between groups (*P* = 0.6). Likewise, the prevalence of inducible ischaemia in individual segments did not differ between LBBB and non-LBBB patients (*Figure 2*).

### Logistic regression

The results of the uni- and multivariate logistic regression analyses are presented in *Tables 3* and *4*.

**Table 3:** Logistic regression for LV non-recovery in LBBB patients

Univariate				Multivariate^a^		
Variable	Odds ratio	95% CI	*P* value	Odds ratio	95% CI	*P* value
Age	1.01	0.98–1.05	0.43			
Female	0.33	1.14–0.77	0.01	0.46	0.19–1.13	0.09
QRS duration	1.02	0.99–1.04	0.11	1.02	0.99–1.04	0.23
Duration between echocardiography and CMR	0.996	0.99–1.0003	0.07			
Pre-scan LVEF	0.92	0.86–0.98	0.013	0.93	0.87–0.99	0.03
Non-ischaemic LGE	1.66	0.61–4.56	0.32			
Ischaemic LGE	2.68	0.73–9.81	0.14			
Inducible ischaemia	0.96	0.18–5.23	0.96			

CMR, cardiac magnetic resonance; LBBB, left bundle branch block; LGE, late gadolinium enhancement; LVEF, left ventricular ejection fraction.

^a^Multivariate regression for pre-scan LVEF, adjusted for sex and QRS duration.

**Table 4:** Logistic regression for LV non-recovery in patients without LBBB

Univariate				Multivariate^a^		
Variable	Odds ratio	95% CI	*P* value	Odds ratio	95% CI	*P* value
Age	1.0048	0.98–1.03	0.65			
Female	0.41	0.24–0.71	0.0014	0.54	0.31–0.95	0.03
QRS duration	1.03	1.008–1.04	0.005	1.02	1.002–1.04	0.03
Duration between echocardiography and CMR	0.99	0.99–1.00	0.041			
Pre-scan LVEF	0.97	0.91–1.0008	0.056			
Non-ischaemic LGE	1.97	1.1–3.52	0.02			
Ischaemic LGE	4.22	1.83–9.75	0.0007	3.95	1.69–9.23	0.0015
Inducible ischaemia	1.52	0.54–4.23	0.36			

CMR, cardiac magnetic resonance; LBBB, left bundle branch block; LGE, late gadolinium enhancement; LVEF, left ventricular ejection fraction.

^a^Multivariate regression for ischaemic LGE, adjusted for sex and QRS duration.

Patients with LBBB lacking LV recovery presented lower LVEF on initial assessment (28.6 ± 7.5 vs. 32.5 ± 5.8%, *P* = 0.01) and reduced LVEF remained an independent predictor of LV non-recovery, even after adjusting for sex and QRS duration (OR 0.93, 95% CI 0.87–0.99, *P* = 0.03). In contrast, the presence of inducible ischaemia and ischaemic and non-ischaemic LGE was not associated with the absence of improvement in LV function in this group.

In comparison, no association between initial LVEF and LV recovery was observed among patients without LBBB. However, the presence of both ischaemic (OR 4.22, 95% CI 1.83 −9.75, *P* = 0.0007) and non-ischaemic LGE (OR 1.97, 95% CI 1.1 −3.52, *P* = 0.02) was indicative of LV non-recovery in this group, and ischaemic LGE remained a predictor of LV non-recovery (OR 3.95, 95% CI 1.69–9.23, *P* = 0.0015) in the multivariate regression analysis.

## Discussion

Our study has several novel findings that may inform the management of patients presenting with heart failure:

1. Patients with HF and LBBB had larger LVEDV and smaller RVEDV than those without LBBB.
2. There was no difference in the prevalence of ischaemic scar, non-ischaemic scar, or inducible ischaemia according to LBBB status.
3. There was no difference in the rate of LV recovery between those with and without LBBB.
4. The presence of ischaemic scar was only predictive of non-recovery of LV function in non-LBBB patients.

To the best of our knowledge, this study provides the largest investigation to date on the recovery of LV function using CMR characterization in HF patients with LBBB who were treated with GDMT.

### The relationship between LBBB and ventricular dilatation

It has been previously suggested that the occurrence of LBBB in individuals with dilated cardiomyopathy (DCM) might exacerbate cardiac dysfunction by inducing further dyssynchrony.^10^ Our study found that LBBB patients had larger LV volumes and smaller RV volumes compared with patients without LBBB. However, there was no difference in LVEF between those with and without LBBB, while patients with LBBB had a higher RVEF. Similarly, a prior investigation documented more dilation of the LV and lesser dilation of the RV in individuals with LBBB.^11^ In contrast to our results, LBBB patients had a greater reduction in LVEF. However, that investigation included patients across the entire range of reduced LVEF while grouping together patients with LBBB and left anterior fascicular block in the analysis.^11^ Nevertheless, it is noteworthy that the characteristics of individuals with mildly reduced LVEF differed from those within more severely reduced LVEF categories.^1^ In patients with LBBB, LVEF is disproportionately decreased relative to the extent of the scar, according to a recent study. This finding suggests that the additional stress induced by LBBB may diminish the effectiveness of compensatory mechanisms.^12^ Hence, it can be challenging to determine if the increased LV volumes in our research are a consequence of dyskinesia resulting from LBBB or of additional wall stress induced by LBBB. However, the imbalance in volumes between the right and left heart, which can result from the septal dyssynchrony secondary to abnormal activation sequence occurring in LBBB, suggests that dyskinesia may be the underlying mechanism.

### Presence and pattern of scar in patients with LBBB and HF

Our investigation revealed that there was no significant difference in prevalence and pattern of both ischaemic and non-ischaemic scars in patients with and without LBBB. Nevertheless, the prevalence of scarring was notably reduced in comparison to previous reports, averaging 46%.^13^

In the overall population of individuals with non-ischaemic DCM, the presence of septal fibrosis is a recognized risk factor for adverse outcomes.^14^ It also appears that individuals exhibiting only LBBB-related dyssynchrony show relatively preserved myocardial deformation compared with those with LGE.^15^ However, the relationship between septal fibrosis and the duration of the QRS complex remains to be defined. While one study failed to establish a correlation between these two parameters,^16^ another smaller study proposed that LBBB patients may have a greater prevalence of septal non-ischaemic LGE, despite exhibiting similar degrees of fibrosis in the remaining segments.^11^ Another recent study examining the relationship between CMR-derived scar and response to CRT also found no difference in the overall scar percentage between patients with or without LBBB. However, in contrast to our results, this research demonstrated that septal scarring was more prevalent in patients with LBBB.^17^ Our work supports the absence of a relationship between left ventricular fibrosis and conduction abnormalities. This was applicable to both segmental fibrosis (including septal fibrosis), as well as overall fibrosis, suggesting that QRS prolongation and ventricular fibrosis are distinct entities in patients with HF.

### LBBB and inducible ischaemia

There is limited evidence in the published literature about the use of myocardial perfusion CMR in patients with LBBB. An experimental investigation including eight dogs revealed that myocardial blood flow in the septum was decreased in LBBB, while increasing in the LV lateral wall. This suggests that ventricular activation during LBBB may cause a redistribution of myocardial blood flow.^18^ However, individuals with DCM and LBBB have equal chances of developing a perfusion defect in the interventricular septum, and the defect may be associated with changes in the myocardial mechanics rather than fibrosis or ischaemia, as demonstrated in a myocardial perfusion SPECT investigation, although this may reflect a technical limitation of SPECT rather than coronary artery disease.^19^ In the current study, perfusion defects by CMR were uncommon in patients with presumed non-ischaemic HF, occurring in only 6.1% of those with LBBB. Additionally, no significant differences were seen between individuals with and without LBBB in terms of global or segmental ischaemia burden. These findings suggest a potential technical limitation of perfusion SPECT that may not be present in perfusion CMR.

### Clinical characteristics of LBBB patients

Consistent with findings from previous investigations, patients with LBBB compared with those without were older and more often female, but had no significant differences in other cardiovascular risk factors (arterial hypertension, hypercholesterolaemia stroke, diabetes, smoking).^11^ The other main difference with evidence from other studies^20^ was that the incidence of AF was lower in patients with LBBB. However, LA dimensions were comparable, and the reasons for variable prevalence of AF are unclear.

### Improvement of LV function in LBBB patients

LVEF is widely recognized as a significant prognostic marker in heart failure patients. The use of GDMT can result in an improvement of LVEF and a reduction in the risk of mortality and hospitalization. While there is inconsistency in the definition of HFimpEF across different studies, it is generally observed that the prognosis of HFimpEF is more favourable regardless of the specific criteria used.^6,21^ The prevalence of HFimpEF we found in patients with LBBB was consistent with that reported in existing literature, which ranged from 10.36% to 52.07%.^6,22–25^

The recovery rate of LBBB patients in our study was comparable to individuals without LBBB. An investigation evaluating patients with LBBB and normal LVEF by echocardiography reported a similar rate of decline in LVEF (32%) as that in our study^26^ although other studies have reported lower rates.^23,27^

The literature presents conflicting data about the impact of GDMT on reverse remodelling in patients with LBBB.^26,28^ Interestingly, our research further demonstrated that LBBB constitutes a distinct phenotype characterized by specific characteristics that can predict LVEF recovery, distinguishing it from patients without LBBB. The only independent determinant to predict LV recovery in patients with LBBB was the initial LVEF, whereas LGE did not demonstrate any predictive value. This agrees with the results of another study that assessed individuals with LBBB and normal LVEF using echocardiography and similarly concluded that a lower baseline LVEF is associated with an increased likelihood of developing reduced LVEF at follow-up.^26^ These findings align with the current guidelines, which propose that CRT should be considered only after 3–6 months of GDMT, rather than supporting the use of early CMR to facilitate more immediate CRT implantation. Hence, this research contributes significant information to the assessment of remodelling and candidate selection for CRT, thereby mitigating the risk of unnecessary device implantation and associated complications in patients who may experience recovery through medical intervention.

In contrast, initial LVEF was not associated with HFimpEF in patients without LBBB, whereas the presence of ischaemic LGE was found to be a predictor of the absence of LVEF recovery in this group. This may indicate that individuals with LBBB exhibit a distinct mechanism of disease progression, presenting as LV dilatation.

### Limitations

Our study had several limitations. First, a smaller number of symptomatic individuals being referred for CMR may lead to a referral bias. Secondly, LVEF was measured by the reporting sonographer rather than in a single laboratory. However, the primary objective of this study was to identify clinical characteristics that could potentially function as indicators of poor LV function recovery rather than comparing LVEF across various modalities. We defined recovery as a categorical variable as rather than a continuous one for the same reason. Thirdly, the absence of ECG data for some of the participants represented another limitation of the study and possible bias. Finally, the participants in this study were real-world patients with HF, and they received individualized treatment using GDMT. Moreover, it should be noted that during our recruitment phase, some of the medical therapies were not yet endorsed by existing recommendations. The majority of patients was receiving therapy with ACE inhibitors and beta blockers, whereas the use of mineralocorticoid receptor antagonists was moderate and that of SGLT2i minimal. The magnitude of LVEF recovery may have been greater if these patients had received the complete recommended therapy for HF and the impact of GDMT on LVEF might warrant further investigation. The period between initial and follow-up assessment was only 70 days and a longer follow-up period may have shown more recovery of LV function. Additionally, the formal quantification of the LGE extent and how it relates to LV recovery could warrant further analysis, in addition to considering the potential advantages of incorporating T1 mapping and ECV.

## Conclusion

In this study, patients presenting with HFrEF and LBBB had larger LV cavities and smaller RV cavities than those without LBBB but no difference in scar or ischaemia. Furthermore, the rates of LV recovery were similar between both groups that supports current guidelines to defer CRT following 3–6 months of GDMT, rather than early CMR and device implantation.

## Consent

This research was approved by the National Research Ethics Committee (17/YH/0300 and 20/NW/0326). Written consent was obtained from every patient.

## Data Availability

Data used in this study are available from the corresponding author on request.

## Lead author biography

Raluca Tomoaia, MD, PhD, holds the position of assistant professor of cardiology at the University of Cluj-Napoca. She focuses on multimodality imaging and holds EACVI certification in transthoracic and transoesophageal echocardiography. Currently, she is a fellow in cardiovascular magnetic resonance imaging at the University of Leeds. A large part of her publications addresses ischaemic heart disease, cardiomyopathies, and valvular heart disease.
